# Performance of ddPCR-GNB for microbial diagnosis of suspected bloodstream infection due to the four most common gram-negative bacteria: a prospective, multicenter study

**DOI:** 10.1128/spectrum.01015-24

**Published:** 2025-02-25

**Authors:** Shan-shan Weng, Ling Lin, Jian-feng Xie, Bang-chuan Hu, Xue-qing Ma, Jiang Xia, Yan Jiang, Hua Zhou, Xiao-yan wu, Yu-hong Jin, Guo-qiu Wu, Yi Yang, Ren-Hua Sun, Yun-song Yu, Dong-dong Zhao

**Affiliations:** 1 Department of Infectious Diseases, Sir Run Run Shaw Hospital, Zhejiang University School of Medicine, Hangzhou, China; 2 Key Laboratory of Microbial Technology and Bioinformatics of Zhejiang Province, Sir Run Run Shaw Hospital, Zhejiang University School of Medicine, Hangzhou, China; 3 Regional Medical Center for National Institute of Respiratory Diseases, Sir Run Run Shaw Hospital, Zhejiang University School of Medicine, Hangzhou, China; 4 Department of Infectious Diseases, Taizhou Hospital of Zhejiang Province, Zhejiang University, Taizhou, China; 5 Jiangsu Provincial Key Laboratory of Critical Care Medicine, Department of Critical Care Medicine, Zhongda Hospital, School of Medicine, Southeast University, Nanjing, China; 6 Intensive Care Unit, Zhejiang Provincial People’s Hospital, People’s Hospital of Hangzhou Medical College, Hangzhou, China; 7 Pilot Gene Technologies (Hangzhou) Co., Ltd, Hangzhou, China; 8 Department of Respiratory and Critical Care Medicine, The First Affiliated Hospital, School of Medicine, Zhejiang University, Hangzhou, China; 9 Department of Laboratory Medicine, The Second Affiliated Hospital of Jiaxing University, Jiaxing, China; 10 Department of Critical Care Medicine, Ningbo Medical Center Lihuili Hospital, Ningbo University, Ningbo, China; 11 Jiangsu Provincial Key Laboratory of Critical Care Medicine, Center of Clinical Laboratory Medicine, Zhongda Hospital, Southeast University, Nanjing, China; Laboratory Corporation of America Holdings, Burlington, North Carolina, USA

**Keywords:** bloodstream infection, rapid diagnosis, droplet digital PCR

## Abstract

We aimed to validate the performance of ddPCR-GNB, a plasma droplet digital PCR panel targeting the four most common gram-negative bacteria, for patients with suspected bloodstream infection (BSI). Patients suspected of having BSIs were prospectively enrolled. The results of blood culture and ddPCR-GNB were compared, and cases with discordant results were arbitrated on the basis of additional microbiological results and clinical evidence. A total of 1,041 patients were enrolled. Blood culture and ddPCR-GNB results were positive for targeted bacteria in 6.3% and 31.7% of patients, respectively. The overall per-patient sensitivity and specificity of ddPCR-GNB for proven BSIs were 98.5% (95% CI, 91.9% to 99.9%) and 72.8% (95% CI, 69.9% to 75.5%), respectively; the negative predictive value was 99.9% (95% CI, 99.2% to 100%). The discordant results included 265 cases (25.5%) with negative companion blood culture results but positive ddPCR-GNB results and one case vice versa. A total of 23.7% of the cases were attributed to probable (*n* = 126) or possible (*n* = 121) BSIs. If both probable and possible BSIs were assumed to be true positives, the per-patient specificity of ddPCR-GNB would be 97.5%. The ddPCR-GNB panel demonstrated excellent microbial diagnostic performance in identifying targeted bacteria for patients with suspected BSI.

**IMPORTANCE:**

This is the first multicentral study to validate the clinical performance of ddPCR in etiological diagnosis of bloodstream infection. The results showed that ddPCR has high sensitivity and increased detection rate compared with blood culture. The study proved the potential of the ddPCR method in microbial diagnoses.

## INTRODUCTION

Bloodstream infection (BSI) is one of the most severe types of infectious diseases caused by pathogenic microorganisms invading the bloodstream and can lead to multiple organ failure, disseminated intravascular coagulation, septic shock, and even death (1–3). BSI and associated sepsis/septic shock always lead to poor outcomes, especially when appropriate antimicrobial therapy and source control are delayed (2). A meta-analysis of the incidence and mortality of adult sepsis revealed that the annual incidence rates of hospital-treated sepsis and severe sepsis were 288 and 148 per 100,000 person-years from 1979 to 2015, and the mortality rates reached 17% and 26%, respectively (4).

Early diagnosis and administration of appropriate antimicrobials are key factors for reducing the high mortality rate of BSIs (2). Blood culture (BC), the current “gold standard” for diagnosing BSIs, usually takes days (5), leading to a significant delay in targeted therapy. Therefore, in addition to BC, developing molecular diagnostic methods that are both rapid and accurate is a top priority. Most of these methods are based on blood culture methods, such as hybridization-based *Quick*FISH, matrix-assisted laser desorption ionization-time of flight mass spectrometry, and FilmArray blood culture identification (6–8), which are still time-consuming. Recently, the T2Bacteria Panel has been proven to be one of the most successful culture-independent molecular diagnostic methods for diagnosing BSIs caused by five common bacteria (9). In addition, digital polymerase chain reaction (dPCR), which is less affected by PCR reaction inhibitors, such as certain blood components, than quantitative PCR (qPCR), is also a promising technique for developing culture-independent molecular diagnostics (10, 11). Droplet dPCR (ddPCR) achieves high sensitivity through quantifying nucleic acid molecules at the single molecule level and has now been used to detect pathogens (10).

According to data from the China Antimicrobial Surveillance Network (12), the top eight causal pathogens of BSIs were *Escherichia coli*, *Klebsiella pneumoniae*, coagulase-negative *Staphylococci*, *Staphylococcus aureus*, *Enterococci*, *Acinetobacter baumannii*, *Pseudomonas aeruginosa*, and *Streptococcus*. In this study, we used a multiplex droplet dPCR panel, named ddPCR-GNB, to detect the four most common gram-negative bacteria (*E. coli*, *K. pneumoniae*, *A. baumannii*, and *P. aeruginosa*), and we aimed to validate the performance of its microbial diagnosis for patients with suspected BSIs.

## MATERIALS AND METHODS

### Study design and participants

This study was a prospective, multicenter study performed to validate the microbial diagnostic application of ddPCR-GNB for suspected BSIs. The study was conducted in four departments of intensive care, hematology, emergency, and infectious diseases from five tertiary hospitals from December 2020 to April 2022.

Patients for whom two sets of diagnostic blood cultures were ordered upon the suspicion of BSIs according to the physician’s discretion and who had sequential organ failure assessment (SOFA) score increases ≥2 points or quick SOFA (qSOFA) score increases of ≥2 points were included. When SOFA and qSOFA scores were temporarily unavailable, patients were also considered eligible when one of the following clinical presentations or laboratory results was met: (i) chills, (ii) fever (body temperature >38.3°C) or hypothermia (body temperature <36.0°C), (iii) WBC >12 × 10^9^ /L or WBC <4 × 10^9^ /L or immature granulocytes >10%, or (iv) PCT >1 ng/mL. Patients with the following conditions were excluded: (i) who did not meet the criteria of suspected BSI, (ii) who refused to draw blood or participate in this study, and (iii) who lacked patient-related clinical information in the electronic medical record system.

None of the included cases were duplicated. The study was approved by the Ethics Committee of our institution (20200818-7), and written informed consent was obtained from each participant or their legal representative.

### Blood culture

Two sets of blood culture bottles (two aerobic bottles and two anaerobic bottles) were collected from two different venipuncture sites for each patient. The volume of blood collected in each bottle was 8–10 mL. Standard-of-care microbiology cultures were obtained at every institution. All the isolates were identified via matrix-assisted laser desorption ionization-time of flight mass spectrometry (Bruker Daltonics, Bremen, Germany).

### Plasma DNA extraction and ddPCR

At least 4 mL of whole blood sample (ethylenediaminetetraacetic acid blood collection tubes) was obtained from one of the venipuncture sites for blood culture testing at the same time, so the blood cultures were defined as companion BCs. The plasma was immediately isolated after centrifugation at 1,600 × *g* and 22°C for 15 min. Plasma DNA extraction was completed within 1 h from 2 mL of plasma via a magnetic serum/plasma DNA kit (TIANGEN Biotech, Beijing, China) and an Auto-Pure 20B nucleic acid purification system (Hangzhou Allsheng Instruments Company, Hangzhou, China) following the manufacturer’s protocol. DNA was eluted in 50 µL of elution buffer and used for the ddPCR assay.

The primers and probes were designed according to the four targeted gram-negative bacteria (*E. coli*, *K. pneumoniae*, *A. baumannii,* and *P. aeruginosa*; Table S1). The ddPCR test was performed via the Droplet Digital PCR System (Pilot Gene Technology, Hangzhou, China) according to the manufacturer’s instructions. Briefly, 5 µL of extracted DNA sample was added to 10 µL of ddPCR premix, which included primers, probes, a dNTP mixture, Taq polymerase, and other necessary components for PCR amplification. The 15 µL mixture was added to a microfluidic chip and loaded into a DG32 droplet generator for droplet generation. The chips were then amplified in a TC1 thermal cycler with the following thermal cycling parameters: 95°C for 5 min, followed by 40 cycles of 95°C for 15 s and 60°C for 30 s. Next, the chips were transferred to a CS5 chip scanner for fluorescence signal reading and data analysis via GenePMS software (v2.0.01.20011). The synthesized target DNA fragment was used as a positive control, and DNase-free water was used as a negative control. A synthesized fragment of porcine herpes virus was used as an internal control.

### Sanger sequencing

In cases where ddPCR-GNB and blood culture results were inconsistent, the gene fragment of the target pathogen was amplified via nested primers and validated via Sanger sequencing (results shown in Table S2). Both rounds of nested PCR were performed in a 20 µL reaction system. The reaction mixture contained 1 µL of template DNA, 0.5 µL of 10 µM forward and reverse primers, and 4 µL of 5 × PCR mixture. The thermal cycle parameters included 95°C for 5 min; 35 cycles of 95°C for 20 s, 58°C for 30 s, and 72°C for 30 s; and a final extension for 5 min at 72°C. The PCR products were analyzed via 1.5% agarose gel electrophoresis and cleaned with a PCR purification kit. Sanger sequencing was performed with the forward and reverse primers of the second round of PCR on an ABI 3730XL DNA Analyzer (Applied Biosystems) via BigDye Terminators V3.1 according to the manufacturer’s instructions.

### Clinical data collection and definitions

Clinical information of the enrolled cases was reviewed and collected, including demographics, underlying diseases, clinical manifestations, antimicrobial treatments, culture results, and patient outcomes at discharge.

“Proven BSI” referred to a positive and consistent companion blood culture result. A “probable BSI” was defined as a negative companion blood culture result but a positive ddPCR-GNB result if the ddPCR-detected microorganism was clinically consistent and was isolated within 15 days from another clinical blood culture specimen or from a specific site (such as the lungs, abdomen, local abscess, or urine). “Possible BSI” was defined as negative companion blood culture results and other supporting culture results but positive ddPCR-GNB results if the detected microorganism was clinically consistent and confirmed by Sanger sequencing. Otherwise, a positive ddPCR-GNB result was considered a “putative false positive.”

Polymicrobial bloodstream infection was defined as a plausible clinical episode in which more than one microorganism was detected by either ddPCR-GNB or blood culture; only the four targeted gram-negative bacteria were evaluated in this study.

### Statistical analysis

The primary outcomes were the sensitivity and specificity of the ddPCR-GNB panel, which were calculated via the “gold standard,” i.e., positive blood culture results (i.e., proven BSI) were used as the reference. Discordant (negative blood culture and positive ddPCR-GNB results or vice versa) cases were further investigated and analyzed according to the criteria of probable, possible, and putative false-positive BSIs defined above.

Since this study detected four types of bacteria, more than one of the four bacteria may be detected in the same patient. Therefore, for the results, we performed data analysis based on the patient-based method and the bacteria-based method, which were expressed as per-patient and per-assay in the text, respectively. For per-patient calculations, each patient’s sample was considered positive or negative based on the results for the four microorganisms in the ddPCR-GNB panel; i.e., if any of the four microorganisms were detected in the sample, it was classified as positive for that specific patient. For the per-assay calculations, the results for individual organisms in each sample were considered separately. The per-assay results of ddPCR are shown in Table S3.

Sensitivity, specificity, positive predictive values (PPV), and negative predictive values (NPV) were calculated according to the following formulas: Sensitivity = Number of true positives / (Number of true positives + Number of false negatives)*100%, Specificity = Number of true negatives / (Number of true negatives + Number of false positives)*100%, PPV = Number of true positives / (Number of true positives + Number of false positives)*100%, and NPV = Number of true negatives / (Number of true negatives + Number of false negatives)*100%.

Statistical analysis was performed with Prism 8.4.3 software (GraphPad Software, San Diego, CA, USA). Continuous variables are expressed as the means and standard deviations (SDs) and were compared through t-tests or Mann‒Whitney tests. Categorical variables are reported as percentages and were compared through the chi-square test. Multiple group comparisons were performed via the Kruskal‒Wallis test. Differences were considered statistically significant if *P* values were ≤0.05.

## RESULTS

### Patient enrollment and BC results

**Fig 1:**
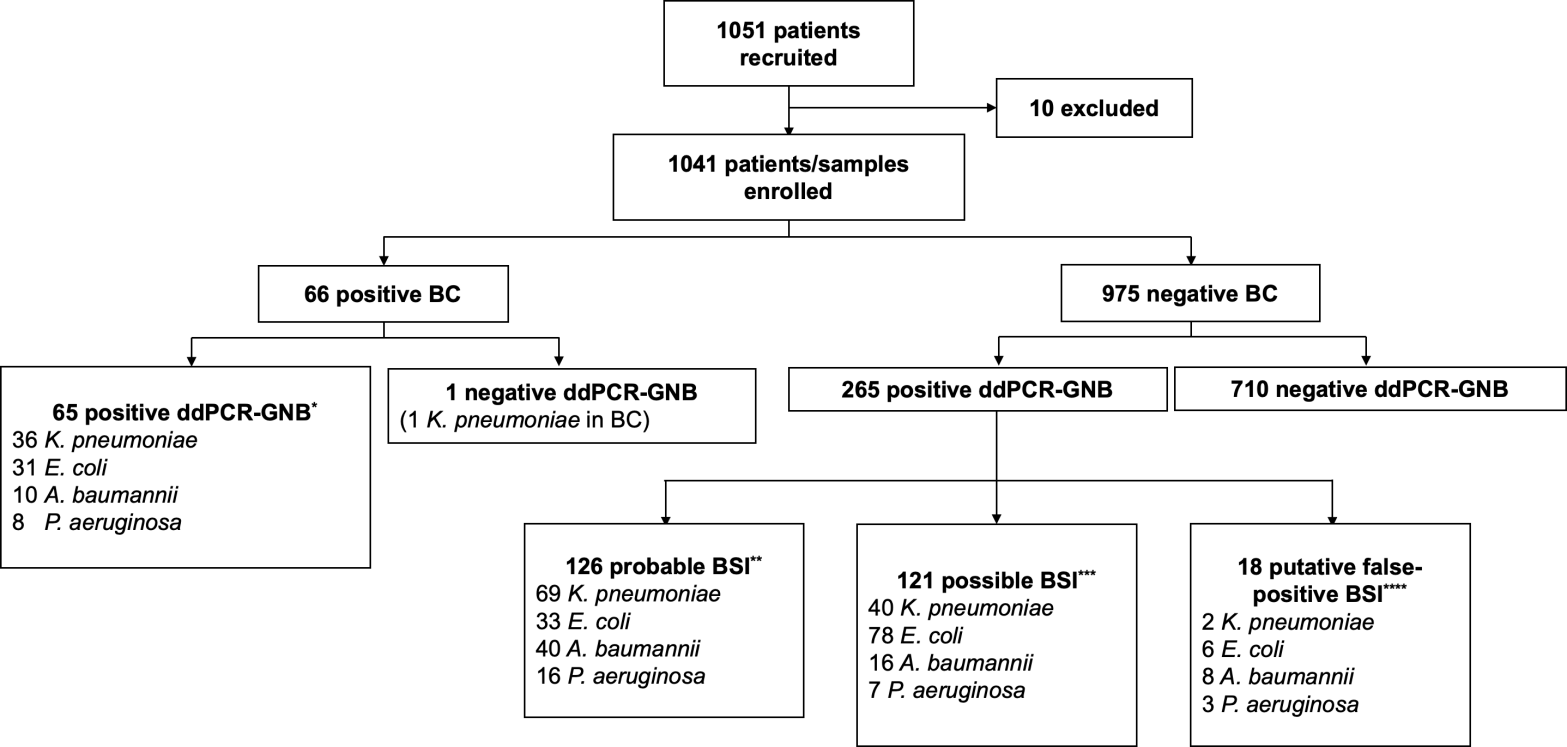
Flowchart of patient enrollment and combined results of blood culture and ddPCR-GNB. * In 12 samples, a second ddPCR-targeted organism was identified that was not identified in the companion BC. In four samples, a third ddPCR-targeted organism was identified. Therefore, 85 organisms were identified in 65 positive samples. ** In 21 samples, a second ddPCR-targeted organism was identified that was not identified in the companion BC. In four samples, a third ddPCR-targeted organism was identified. In one sample, a fourth ddPCR-targeted organism was identified. Therefore, 158 organisms were identified in 126 positive samples. *** In 18 samples, a second ddPCR-targeted organism was identified that was not identified in the companion BC. In one sample, a third ddPCR-targeted organism was identified. Therefore, 141 organisms were identified in 121 positive samples. **** In one sample, a second ddPCR-targeted organism was identified that was not identified in the companion BC. Therefore, 19 organisms were identified in 18 positive samples.

A total of 1,051 patients were consecutively recruited from December 2020 to April 2022, and 10 patients were excluded, resulting in the final enrollment of 1,041 patients in the study (Fig. 1).

The mean patient age was 63.4 ± 16.1 years, and 65.0% (677 of 1,041) were men. Most patients were enrolled in the intensive care unit (73.0%), followed by the infectious diseases department (12.7%). A significant increase in the inflammatory index was observed, with average values of 11.0 ng/ml for procalcitonin and 136.4 mg/L for C-reactive protein. The majority (70.9%) showed improvement or stability at discharge, whereas others experienced deterioration or death (Table 1).

**TABLE 1:** Patient demographics and clinical characteristics

Characteristics	Value
Age at diagnosis (mean ± SD)	63.4 ± 16.1
Male	677 (65.0%)
Antibiotic treatment before sampling	913 (87.7%)
Ward	
Intensive care unit	760 (73.0%)
Infectious diseases department	132 (12.7%)
Emergency department	80 (7.7%)
Hematology department	69 (6.6%)
Laboratory findings (mean ± SD)	
Procalcitonin (ng/mL)	11.0 ± 20.9
C-reactive protein (mg/L)	136.4 ± 89.0
White blood cell count (10^9^ /L)	12.9 ± 16.5
Neutrophil percentage (%)	82.2 ± 17.5
Clinical outcome at discharge	
Improved or stable	738 (70.9%)
Deteriorated or dead	296 (28.4%)

In general, blood cultures were positive in 10.3% (107 of 1,041) of the patients (Table 2). Among them, the microorganisms included in the ddPCR-GNB panel were identified in 66 patients (6.3% of all patients), and only 1 patient was positive for multiple bacteria (*K. pneumoniae* and *A. baumannii*). The most frequently isolated bacterium in BC was *K. pneumoniae* (*n* = 33). The untargeted isolates are listed in Table S4. Only three strains of *S. aureus* were isolated. Coagulase-negative staphylococci were recovered from 10.3% (11 of 107) of positive blood cultures, a substantial portion of which were classified as contaminants according to routine clinical experience.

**TABLE 2:** Positive results from BC or ddPCR-GNB^*f*^

Species (no. of isolates identified)	ddPCR-GNB	BC	Both
*K. pneumoniae* (147)	147	33	32^*d*^
*E. coli* (148)	148	22	22
*A. baumannii* (74)	74	7	7
*P. aeruginosa* (34)	34	5	5
Others (25)	NA	45^*a*^	NA
Total	403^*b*^	112^*c*^	66^*e*^

^*a*^ Forty-five organisms in 42 samples, with a single organism detected in 39 samples and 2 organisms detected in 3 samples.

^*b*^ A total of 403 organisms were detected in 330 samples, with a single organism detected in 268 samples, 2 organisms detected in 52 samples, 3 organisms detected in 9 samples, and 4 organisms detected in 1 sample.

^*c*^ A total of 112 organisms were detected in 107 samples, with a single organism detected in 102 samples and 2 organisms detected in 5 samples. In one sample, one organism (Eci) within the detection spectrum of ddPCR-GNB and one organism (*Gemella morbillorum*) outside the spectrum were both detected. In one sample, both Kp and Ab were detected.

^*d*^ In one sample, BC was positive, whereas ddPCR-GNB was negative.

^*e*^ In one sample, BC and ddPCR-GNB both detected two bacteria, *K. pneumoniae* and *A. baumannii*. Thus, the results of 66 tests in 65 patients were consistent.

^*f*^ NA: Not applicable.

### Microbial performance and clinical significance of the ddPCR-GNB panel

In our study, 31.7% (330/1,041) of the samples reported positive by ddPCR-GNB, and a total of 403 bacterial species were identified (Table 2), which is 6 times the number detected by blood culture. Among the positive ddPCR-GNB results, *K. pneumoniae* (*n* = 147) and *E. coli* (*n* = 148) were most frequently detected, followed by *A. baumannii* (*n* = 74) and *P. aeruginosa* (*n* = 34).

The performance of ddPCR-GNB, with proven BSI caused by the targeted bacteria as the reference, is summarized in Table 3. Both per-patient and per-assay analyses were conducted. The first four columns of data are calculated based on per-assay, showing the detection efficacy of ddPCR for the four bacteria, and the last column is calculated based on per-patient, showing the overall detection efficacy. The combined results of blood culture and ddPCR-GNB are shown in Fig. 1.

**TABLE 3:** Performance of ddPCR-GNB for the diagnosis of proven BSIs caused by targeted bacteria

	ddPCR-GNB results by detection channel				
	**Kp**	**Eci**	**Ab**	**Pa**	**Total**
Matched positives, n	32	22	7	5	65^a^
Matched negatives, n	893	893	967	1,007	710
ddPCR extra detections, n	115	126	67	29	265
ddPCR misses, n	1	0	0	0	1
Overall agreement, %	88.9	87.9	93.6	97.2	74.5
Sensitivity, %	97.0	100	100	100	98.5
Specificity, %	88.6	87.6	93.5	97.2	72.8
PPV, %	21.8	14.9	9.5	14.7	19.7
NPV, %	99.9	100	100	100	99.9

^*a*^ In one sample, both BC and ddPCR-GNB detected two organisms (Kp and Ab).

BC and ddPCR-GNB results were both positive in 65 patients, both negative in 710 patients, and discordant in 266 patients. The level of agreement between BC and ddPCR-GNB was 74.5% (775/1,041). The overall sensitivity and specificity of ddPCR-GNB were 98.5% (65/66) (95% CI, 91.9% to 99.9%) and 72.8% (710/975) (95% CI, 69.9% to 75.5%), respectively. The positive predictive value and negative predictive value of ddPCR-GNB were 19.7% (95% CI, 15.8% to 24.3%) and 99.9% (95% CI, 99.2% to 100%), respectively. For per-assay analyses, the sensitivities of ddPCR-GNB were 97.0% (32/33) (95% CI, 84.7% to 99.8%), 100% (22/22) (95% CI, 85.1% to 100%), 100.00% (7/7) (95% CI, 64.6% to 100%), and 100% (5/5) (95% CI, 56.6% to 100%) for *K. pneumoniae*, *E. coli*, *A. baumannii*, and *P. aeruginosa*, respectively. The specificities were 88.6% (893/1008) (95% CI, 86.5% to 90.4%), 87.6% (893/1019) (95% CI, 85.5% to 89.5%), 93.5% (967/1034) (95% CI, 91.9% to 94.9%), and 97.2% (1007/1036) (95% CI, 96.0% to 98.0%) for *K. pneumoniae*, *E. coli*, *A. baumannii*, and *P. aeruginosa*, respectively.

To explore the clinical significance of ddPCR-GNB, the main inflammatory indicators, antimicrobials before sampling, and patient outcomes at discharge were compared between patients with positive ddPCR-GNB results and those with negative results (Table S5). The indices of patients with positive BC results and negative results were also compared via a similar approach. Procalcitonin and neutrophil percentages were significantly greater in the positive control groups for both BC and ddPCR-GNB. There was a lower percentage (80.3% versus 88.8%) of antimicrobials before sampling in the BC-positive group, while no significant difference was found between the ddPCR-GNB groups. A lower percentage (64.0% versus 74.8%) of positive outcomes was found in the ddPCR-GNB-positive group; in contrast, no significant difference was found between the BC groups.

### Evaluation of discordancy between blood culture and ddPCR-GNB

There were 265 (25.5%) patients with negative blood cultures and positive ddPCR-GNB results. To evaluate these cases, additional microbiological and clinical evidence were analyzed. All 265 samples were subjected to Sanger sequencing, and the results are shown in Table S2. Combined with the Sanger sequencing results and the culture results of other sites, a total of 126 (47.5%) patients met the criteria for probable BSI, 121 (45.7%) met the criteria for possible BSI, and the remaining 18 patients (6.8%) were presumptive false positives (Fig. 1; Table 4).

**TABLE 4:** Arbitration of ddPCR-GNB results with composite evaluation

	ddPCR-GNB results by composite evaluation		
	Proven	Plus probable	Plus possible
Matched positives, n	65^*a*^	191	312
Matched negatives, n	710	710	710
ddPCR extra detections, n	265	139	18
ddPCR misses, n	1	1	1
Overall agreement, %	74.5	86.6	98.2
Sensitivity, %	98.5	99.5	99.7
Specificity, %	72.8	83.6	97.5
PPV, %	19.7	57.9	94.6
NPV, %	99.9	99.9	99.9

^*a*^ In one sample, both BC and ddPCR-GNB detected two organisms (Kp and Ab).

Taking both proven and probable BSIs as references, the sensitivity and specificity of ddPCR-GNB would have been 99.5% (95% CI, 97.1% to 100%) and 83.6% (95% CI, 81.0% to 86.0%), whereas the per-patient specificity would have increased to 97.5% (95% CI, 96.1% to 98.4%) if both probable and possible BSIs had been assumed to be true positives (Table 4).

Only one patient had a positive BC result but a negative ddPCR-GNB result. The blood culture results reported *Klebsiella pneumoniae*, while contemporary plasma metagenomic next-generation sequencing (mNGS) testing revealed *Klebsiella variicola* positivity. The genomic DNA of the isolated strain from the patient was extracted for PCR amplification separately via two pairs of primers designed according to the genomes of the two species, i.e., *K. variicola* and *K. pneumoniae*. Only primers designed according to *K. variicola* yielded positive results, and subsequent Sanger sequencing confirmed the detection of *K. variicola*.

Among the 330 patients with positive ddPCR-GNB results, 18.8% (62/330) had multiple pathogens detected (Table S4). There were 52/62 (83.9%) polymicrobial infections with 2 of the 4 targeted bacteria, 9/62 (14.5%) with 3 species, and 1/62 (1.6%) with 4 species. In contrast, only one case of polymicrobial bloodstream infection, involving cocultivation of *K. pneumoniae* and *A. baumannii*, was identified by blood culture.

## DISCUSSION

BSI is a serious, life-threatening condition and poses a significant burden in terms of morbidity and mortality worldwide (2). Prompt and precise pathogen detection is crucial for the diagnosis and management of BSIs. T2 magnetic resonance, qPCR, dPCR, and mNGS all exhibit considerable promise as culture-independent molecular techniques that are currently undergoing rigorous development and validation (13, 14). Compared with qPCR, dPCR is less affected by PCR inhibitors and less affected by host DNA compared with mNGS. ddPCR is a method for performing dPCR that is based on water‒oil emulsion droplet technology. A sample is fractionated into thousands of droplets, and PCR amplification of the template molecules occurs in each individual droplet. Therefore, this technique is highly reproducible and can achieve absolute quantification without the need for a standard curve (10, 11). Hu et al. reported that ddPCR may offer advantages in terms of detection velocity and heightened sensitivity within the defined range of detection (15). Thus, we developed ddPCR-GNB, a panel targeting the four most common gram-negative bacteria involved in bloodstream infections based on a plasma ddPCR test.

In this multicenter study, 1,041 patients were included to validate ddPCR-GNB. Sixty-five patients were categorized as having proven BSIs confirmed by at least one positive blood culture, and 330 patients tested positive for ddPCR-GNB. The results revealed a sensitivity and specificity of 98.5% and 72.8%, respectively. The NPV in this cohort approached 99.9%. Composite microbiological and clinical criteria were utilized to resolve any discrepancies. This involved considering results from multiple sources, such as blood cultures taken at different time points or culture results from other sites, in addition to plasma Sanger sequencing. The specificity increased to 83.6% when probable BSIs were considered true positives that were not identified by companion blood cultures and to 97.5% when both probable and possible BSIs were considered true positives. However, the definitions of possible and probable BSIs should be further explored, and the results should be deduced with caution. Notably, the ddPCR-GNB results were positive in 4.7-fold more patients with proven, probable, or possible BSIs (*n* = 312) than in those with positive blood cultures (*n* = 66).

In this study, the positivity of ddPCR-GNB was much greater than that of blood culture. We found that 6.3% of patients had positive targeted blood cultures and that 31.7% of patients had positive ddPCR-GNB results. The lower antibiotic usage before sampling in the proven group (Table S6) indicates that the positivity of BC is significantly affected by prior exposure to antimicrobials. A positive ddPCR-GNB result with negative companion blood cultures could be explained by the presence of viable but nonculturable pathogens after antimicrobial exposure that would otherwise be missed by blood culture (16) or by the fact that circulating cell-free DNA molecules originate from dying cells (17) and that the number of genome copies is much greater than the number of bacteria circulating during bloodstream infections (18). The application of ddPCR for the diagnosis of bacterial infections via the detection of cell-free bacterial DNA has been reported in several preliminary single-center studies. All these studies have consistently demonstrated the superior detection efficacy of ddPCR over blood culture methods (19–21).

The question of whether the bacteria detected by ddPCR-GNB are false positive or a true reflection of the causative microorganisms missed by blood culture continues to be a point of debate. Indeed, the detection of microbial DNA cannot be guaranteed; however, in this cohort, the arrangement of two sets of blood cultures according to physician discretion and the high prevalence of antimicrobial treatment indicate valid concerns of active infection. Furthermore, Sanger sequencing confirmed the authenticity of the nucleic acid detection results. With the composite evaluation of additional microbiological results and clinical evidence, we were able to resolve discordant cases.

Furthermore, our study suggests that patients with positive ddPCR results might have a worse prognosis. We speculate that, in addition to providing a microbiological diagnosis, ddPCR results may reflect the severity of infection. This speculation remains to be further investigated. This can be an epiphenomenon that reflects the overall disease burden, and microbial DNA itself can result in an inflammatory cascade. The epiphenomenon can be related to leakage of microbial contents from a porous gastrointestinal tract as an indication of bacterial translocation (22).

The prevalence of polymicrobial etiology in patients with BSI has been reported to be 6%–32%, which is independently associated with high mortality (23–25). In our study, among the 66 patients whose blood cultures were positive, only one had multiple pathogens detected. However, among the 330 patients who were ddPCR-GNB positive, 18.8% (62/330) had multiple pathogens detected. Although this phenomenon cannot directly prove that multiple pathogens detected by ddPCR correspond to multiple infections, it suggests that ddPCR has the ability to detect multiple pathogens simultaneously. Similar results have been reported previously; specifically, blood culture and ddPCR were positive in 16.2% (71 of 438) and 41.1% (180 of 438) of episodes, respectively, and polymicrobial bloodstream infections were detected in 8.0% (6 of 71) and 38.3% (69 of 180) of blood culture and ddPCR, respectively (11). This suggested that ddPCR may be more clinically efficient than blood culture in detecting polymicrobial bloodstream infections (14).

Several limitations should be highlighted in this study. First, the ddPCR-GNB panel covered only the four most common gram-negative bacteria, and even genetically closely related bacteria could be missed, resulting in a false-negative report. Second, plasma cell-free DNA was targeted in the test, and the clinical significance of bacterial cell-associated DNA and cell-free DNA should be determined by further investigations. Finally, since the four targeted gram-negative bacteria constituted a significant proportion of the causative microorganisms and the empirical usage of antimicrobial drugs was prevalent in this cohort, caution should be exercised when extrapolating the conclusions drawn from the study.

### Conclusion

In conclusion, ddPCR-GNB demonstrates excellent microbial diagnostic performance in patients with suspected BSI. It certainly excels in detecting polymicrobial infections and may offer additional information with which to evaluate disease severity. This assay is not intended to replace culture methods, and additional studies need to be carried out to further assess its diagnostic value and clinical impact.

## Data Availability

All the data generated or analyzed during this study are included in this published article.

## SUPPLEMENTAL MATERIAL

Supplemental tables spectrum.01015-24-s0001.docx Tables S1 to S6.

ASM does not own the copyrights to Supplemental Material that may be linked to, or accessed through, an article. The authors have granted ASM a non-exclusive, world-wide license to publish the Supplemental Material files. Please contact the corresponding author directly for reuse.

## References

[1] Kern WV, Rieg S. 2020. Burden of bacterial bloodstream infection-a brief update on epidemiology and significance of multidrug-resistant pathogens. Clin Microbiol Infect 26:151–157. doi:10.1016/j.cmi.2019.10.031 31712069

[2] Timsit JF, Ruppé E, Barbier F, Tabah A, Bassetti M. 2020. Bloodstream infections in critically ill patients: an expert statement. Intensive Care Med 46:266–284. doi:10.1007/s00134-020-05950-6 32047941 PMC7223992

[3] Iba T, Levi M, Levy JH. 2020. Sepsis-induced coagulopathy and disseminated intravascular coagulation. Semin Thromb Hemost 46:89–95. doi:10.1055/s-0039-1694995 31443111

[4] Fleischmann C, Scherag A, Adhikari NKJ, Hartog CS, Tsaganos T, Schlattmann P, Angus DC, Reinhart K. 2016. Assessment of global incidence and mortality of hospital-treated sepsis. current estimates and limitations. Am J Respir Crit Care Med 193:259–272. doi:10.1164/rccm.201504-0781OC 26414292

[5] Hall KK, Lyman JA. 2006. Updated review of blood culture contamination. Clin Microbiol Rev 19:788–802. doi:10.1128/CMR.00062-05 17041144 PMC1592696

[6] Kothari A, Morgan M, Haake DA. 2014. Emerging technologies for rapid identification of bloodstream pathogens. Clin Infect Dis 59:272–278. doi:10.1093/cid/ciu292 24771332 PMC4368854

[7] Simon L, Ughetto E, Gaudart A, Degand N, Lotte R, Ruimy R. 2019. Direct Identification of 80 percent of bacteria from blood culture bottles by matrix-assisted laser desorption ionization-time of flight mass spectrometry using a 10-minute extraction protocol. J Clin Microbiol 57:e01278-18. doi:10.1128/JCM.01278-18 30463897 PMC6355546

[8] Opota O, Croxatto A, Prod’hom G, Greub G. 2015. Blood culture-based diagnosis of bacteraemia: state of the art. Clin Microbiol Infect 21:313–322. doi:10.1016/j.cmi.2015.01.003 25753137

[9] Nguyen MH, Clancy CJ, Pasculle AW, Pappas PG, Alangaden G, Pankey GA, Schmitt BH, Rasool A, Weinstein MP, Widen R, Hernandez DR, Wolk DM, Walsh TJ, Perfect JR, Wilson MN, Mylonakis E. 2019. Performance of the T2bacteria panel for diagnosing bloodstream infections: a diagnostic accuracy study. Ann Intern Med 170:845–852. doi:10.7326/M18-2772 31083728

[10] Chen B, Jiang Y, Cao X, Liu C, Zhang N, Shi D. 2021. Droplet digital PCR as an emerging tool in detecting pathogens nucleic acids in infectious diseases. Clinica Chimica Acta 517:156–161. doi:10.1016/j.cca.2021.02.008 33662358

[11] Wu J, Tang B, Qiu Y, Tan R, Liu J, Xia J, Zhang J, Huang J, Qu J, Sun J, Wang X, Qu H. 2022. Clinical validation of a multiplex droplet digital PCR for diagnosing suspected bloodstream infections in ICU practice: a promising diagnostic tool. Crit Care 26:243. doi:10.1186/s13054-022-04116-8 35941654 PMC9358819

[12] Hu F, Yuan L, Yang Y, Xu Y, Huang Y, Hu Y, Ai X, Zhuo C, Su D, Shan B, Du Y, Yu Y, Lin J, Sun Z, Chen Z, Xu Y, Zhang X, Wang C, He L, Ni Y, Zhang Y, Lin D, Zhu D, Zhang Y. 2022. A multicenter investigation of 2,773 cases of bloodstream infections based on China antimicrobial surveillance network (CHINET). Front Cell Infect Microbiol 12:1075185. doi:10.3389/fcimb.2022.1075185 36590586 PMC9798236

[13] Peker N, Couto N, Sinha B, Rossen JW. 2018. Diagnosis of bloodstream infections from positive blood cultures and directly from blood samples: recent developments in molecular approaches. Clin Microbiol Infect 24:944–955. doi:10.1016/j.cmi.2018.05.007 29787889

[14] Doualeh M, Payne M, Litton E, Raby E, Currie A. 2022. Molecular methodologies for improved polymicrobial sepsis diagnosis. Int J Mol Sci 23:4484. doi:10.3390/ijms23094484 35562877 PMC9104822

[15] Hu B, Tao Y, Shao Z, Zheng Y, Zhang R, Yang X, Liu J, Li X, Sun R. 2021. A comparison of blood pathogen detection among droplet digital PCR, metagenomic next-generation sequencing, and blood culture in critically Ill patients with suspected bloodstream infections. Front Microbiol 12:641202. doi:10.3389/fmicb.2021.641202 34079528 PMC8165239

[16] Fleischmann S, Robben C, Alter T, Rossmanith P, Mester P. 2021. How to evaluate non-growing cells-current strategies for determining antimicrobial resistance of VBNC bacteria. Antibiotics (Basel) 10:115. doi:10.3390/antibiotics10020115 33530321 PMC7912045

[17] Blauwkamp TA, Thair S, Rosen MJ, Blair L, Lindner MS, Vilfan ID, Kawli T, Christians FC, Venkatasubrahmanyam S, Wall GD, Cheung A, Rogers ZN, Meshulam-Simon G, Huijse L, Balakrishnan S, Quinn JV, Hollemon D, Hong DK, Vaughn ML, Kertesz M, Bercovici S, Wilber JC, Yang S. 2019. Analytical and clinical validation of a microbial cell-free DNA sequencing test for infectious disease. Nat Microbiol 4:663–674. doi:10.1038/s41564-018-0349-6 30742071

[18] Opota O, Jaton K, Greub G. 2015. Microbial diagnosis of bloodstream infection: towards molecular diagnosis directly from blood. Clin Microbiol Infect 21:323–331. doi:10.1016/j.cmi.2015.02.005 25686695

[19] Ziegler I, Lindström S, Källgren M, Strålin K, Mölling P. 2019. 16S rDNA droplet digital PCR for monitoring bacterial DNAemia in bloodstream infections. PLoS ONE 14. doi:10.1371/journal.pone.0224656 PMC6853374 31721817

[20] Wouters Y, Dalloyaux D, Christenhusz A, Roelofs HMJ, Wertheim HF, Bleeker-Rovers CP, Te Morsche RH, Wanten GJA. 2020. Droplet digital polymerase chain reaction for rapid broad-spectrum detection of bloodstream infections. Microb Biotechnol 13:657–668. doi:10.1111/1751-7915.13491 31605465 PMC7111091

[21] Shao Z, Zhu J, Wei Y, Jin J, Zheng Y, Liu J, Zhang R, Sun R, Hu B. 2022. Pathogen load and species monitored by droplet digital PCR in patients with bloodstream infections: a prospective case series study. BMC Infect Dis 22:771. doi:10.1186/s12879-022-07751-2 36195855 PMC9531393

[22] O’Dwyer MJ, Starczewska MH, Schrenzel J, Zacharowski K, Ecker DJ, Sampath R, Brealey D, Singer M, Libert N, Wilks M, Vincent J-L. 2017. The detection of microbial DNA but not cultured bacteria is associated with increased mortality in patients with suspected sepsis-a prospective multi-centre European observational study. Clin Microbiol Infect 23:208. doi:10.1016/j.cmi.2016.11.010 27890455

[23] Pammi M, Zhong D, Johnson Y, Revell P, Versalovic J. 2014. Polymicrobial bloodstream infections in the neonatal intensive care unit are associated with increased mortality: a case-control study. BMC Infect Dis 14:390. doi:10.1186/1471-2334-14-390 25022748 PMC4226990

[24] Yo C-H, Hsein Y-C, Wu Y-L, Hsu W-T, Ma MH-M, Tsai C-H, Chen S-C, Lee C-C. 2019. Clinical predictors and outcome impact of community-onset polymicrobial bloodstream infection. Int J Antimicrob Agents 54:716–722. doi:10.1016/j.ijantimicag.2019.09.015 31560960

[25] Lin J-N, Lai C-H, Chen Y-H, Chang L-L, Lu P-L, Tsai S-S, Lin H-L, Lin H-H. 2010. Characteristics and outcomes of polymicrobial bloodstream infections in the emergency department: a matched case-control study. Acad Emerg Med 17:1072–1079. doi:10.1111/j.1553-2712.2010.00871.x 21040108

