# Supplementary material for: Performance of ddPCR-GNB for microbial diagnosis of suspected bloodstream infection due to the four most common gram-negative bacteria: a prospective, multicenter study
Source: Microbiol Spectr. 2025 Feb 25;13(4):e01015-24. doi: 10.1128/spectrum.01015-24 (PMC11960046; doi:10.1128/spectrum.01015-24)
Supplement: Supplemental tables — Tables S1 to S6. [file spectrum.01015-24-s0001.docx]

**Table S1 Primers for ddPCR-GNB**

| **Organism** | **Primers sequence (5’-3’)** |
| --- | --- |
| *A. baumannii* | AB-F: GCAACGCGAAGAACCTTACC |
|  | AB-R: GTTCCCGAAGGCACCAATC |
| *E. coli* | EC-F: GGCCTTCGGGTTGTAAAGTAC |
|  | EC-R: TCTTCTGCGGGTAACGTCAAT |
| *K. pneumonia* | KP-F: AGCCTGATGCAGCCATGC |
|  | KP-R: TTAGCCGGTGCTTCTTCTGC |
| *P. aeruginosa* | PA-F: GCAACGCGAAGAACCTTACC |
|  | PA-R: GTTCCCGAAGGCACCAATC |

**Table S2 The Sanger sequencing results of 265 cases with negative BC results and positive ddPCR-GNB results**

| **Species** | **ddPCR-GNB** |  | **Sanger sequencing** | | |
| --- | --- | --- | --- | --- | --- |
|  |  | **consistent** | | **inconsistent** | **negative** |
| *K. pneumoniae* | 73 | 71 | | 0 | 2 |
| *E. coli* | 88 | 87 | | 0 | 1 |
| *A. baumannii* | 44 | 42 | | 0 | 2 |
| *P. aeruginosa* | 14 | 11 | | 0 | 3 |
| *Kp and Eci* | 20 | 20 | | 0 | 0 |
| *Kp and Ab* | 7 | 6 | | 1* | 0 |
| *Kp and Pa* | 5 | 4 | | 1** | 0 |
| *Eci and Ab* | 6 | 5 | | 0 | 1 |
| *Ab and Pa* | 2 | 2 | | 0 | 0 |
| *Kp, Eci and Ab* | 1 | 1 | | 0 | 0 |
| *Kp, Eci and Pa* | 1 | 1 | | 0 | 0 |
| *Kp, Ab and Pa* | 3 | 0 | | 3*** | 0 |
| *Kp, Eci, Ab and Pa* | 1 | 1 | | 0 | 0 |
| Total | 265 | 251 | | 5 | 9 |

**Kp* detected with *Ab* not detected

***Kp* detected with *Pa* not detected

*** In 2 cases, *Pa* was not detected, with *Kp* and *Ab* detected; in the other 1 case, *Ab* was not detected, with *Kp* and *Pa* detected.

**Table S3 The per-assay results of ddPCR**

|  | *Kp* | *Eci* | *Ab* | *Pa* |
| --- | --- | --- | --- | --- |
| proven BSI | 32 | 22 | 7 | 5 |
| probable BSI | 67 | 29 | 38 | 14 |
| possible BSI | 46 | 94 | 25 | 14 |
| putative false-positive | 2 | 3 | 4 | 1 |

**Table S4 Bacterial Species Identified by BC and ddPCR-GNB**

| Bacterial Species Identified by  BC and ddPCR-GNB | Positive ddPCR-GNB | Positive BC |
| --- | --- | --- |
| ddPCR-GNB targeted |  |  |
| *Klebsiella pneumoniae* | 95 | 32 |
| *Escherichia coli* | 108* | 22* |
| *Acinetobacter baumannii* | 49 | 6 |
| *Pseudomonas aeruginosa* | 16 | 5 |
| *K pneumoniae and A baumannii* | 9 | 1 |
| *K pneumoniae and E coli* | 26 | NA |
| *K pneumoniae and P aeruginosa* | 7 | NA |
| *E coli and A baumannii* | 7 | NA |
| *E coli and P aeruginosa* | 1 | NA |
| *A baumannii and P aeruginosa* | 2 | NA |
| *K pneumoniae, E coli and A baumannii* | 2 | NA |
| *K pneumoniae, A baumannii and P aeruginosa* | 4 | NA |
| *K pneumoniae, E coli and P aeruginosa* | 3 | NA |
| *K pneumoniae, E coli, A baumannii and P aeruginosa* | 1 | NA |
| Other bacteria |  |  |
| *Staphylococcus aureus* | NA | 3 |
| *Coagulase-negative Staphylococcus Streptococcus species* | NA | 11 |
| *Enterococcus faecium* | NA | 6 |
| *Corynebacterium striatum* | NA | 1 |
| *Streptococcus pneumoniae* | NA | 1 |
| *Streptococcus mitis* | NA | 2 |
| *Gemella mobillorum* | NA | 1** |
| *Stenotrophomonas maltophilia* | NA | 4 |
| *Proteus mirabilis* | NA | 2 |
| *Salmonella enterica* | NA | 1 |
| *Vibrio vulnificus* | NA | 1 |
| Yeasts |  |  |
| *Candida species* | NA | 3 |
| *Cryptococcus neoformans* | NA | 2 |
| *Trichosporon asahii* | NA | 1 |
| Polymicrobial |  |  |
| *Streptococcus anginosus and Bacteroides fragilis* | NA | 1 |
| *Enterobacter cloacae and Fusobacterius varium* | NA | 1 |
| *Staphylococcus epidermidis and  Candida tropicalis* | NA | 1 |

* Included is 1 sample in which both Eci and Gemella mobillorum were detected by BC and Eci was detected by ddPCR-GNB.

** In this 1 sample, Gemella mobillorum and Eci were detected in BC synchronously.

**Table S5 Characteristics of the ddPCR-GNB-positive and ddPCR-GNB-negative groups**

|  | **Positive**  **ddPCR-GNB (n=330)** | **Negative**  **ddPCR-GNB (n=711)** | **P value** | **Positive**  **BC (n=66)** | **Negative**  **BC (n=975)** | **P value** |
| --- | --- | --- | --- | --- | --- | --- |
| PCT (ng/mL) | 19.2 | 7.1 | <0.05 | 23.8 | 10.1 | <0.05 |
| CRP (mg/L) | 162.7 | 124.0 | <0.05 | 144.9 | 135.9 | 0.45 |
| WBC (10^9^/L) | 12.4 | 13.2 | 0.46 | 13.3 | 12.9 | 0.86 |
| N% (%) | 85.7 | 80.6 | <0.05 | 87.5 | 81.9 | <0.05 |
| Antibiotics before sampling | 89.6% | 87.6% | 0.35 | 80.3% | 88.8% | <0.05 |
| Improved or stable outcome | 64.0% | 74.8% | <0.05 | 69.2% | 71.5% | 0.67 |

**Table S6 Antibiotic treatment before sampling among patients with positive ddPCR-GNB results**

|  | Proven (n=65) | Probable (n=126) | Possible (n=121) | P value |
| --- | --- | --- | --- | --- |
| Antibiotics before sampling | 80.0% | 95.2% | 91.7% | <0.05 |
